# Strengthening Pandemic Preparedness Through Noncommunicable Disease Strategies

**DOI:** 10.5888/pcd18.210237

**Published:** 2021-10-21

**Authors:** Deliana A. Kostova, Ronald L. Moolenaar, Gretchen Van Vliet, Ally Lasu, Michael Mahar, Patricia Richter

**Affiliations:** 1 Division of Global Health Protection, Centers for Disease Control and Prevention, 1600 Clifton Rd, Atlanta, Georgia; 2 RTI International, Research Triangle Park, North Carolina

The COVID-19 pandemic has demonstrated the effect of noncommunicable diseases (NCDs) on infectious disease outcomes. This effect can be observed at both the individual and the population level. At the individual level, the presence of preexisting chronic conditions increases a patient’s risk of severe COVID-19 disease (1). At the population level, the aggregate prevalence of chronic conditions could compound the pandemic’s overall burden on health systems and the economy (2).

Given the role of NCDs in infectious disease and pandemic outcomes, future pandemic preparedness plans could be improved by incorporating selected NCD-related objectives to complement the plans’ core focus on infectious diseases. In a previous publication, we presented a conceptual framework that outlined the points of convergence among infectious disease outbreaks, pandemic preparedness, and NCDs (3). We ascertained that NCDs play a role in 4 factors that determine the course of infectious disease outbreaks: the host population, the disease agent, the physical environment, and the social environment. First, the increased prevalence of NCDs in a host population raises its susceptibility to outbreaks, while also playing into a reverse feedback loop where infection in an individual patient can independently generate NCD sequelae, requiring treatment of both. Second, the presence of NCDs and their risk factors can exacerbate the pathogenicity of the infectious disease agent. Third, the prevalence of NCDs shapes the physical environment by steering health systems toward primary care, where the resulting infrastructure can support pandemic response by increasing staffing and supplementing supply chains. Fourth, the prevalence of NCDs affects the social environment by driving health care budgets and providing avenues for sustainable financing. In summary, the multipronged role of NCDs in infectious disease outbreaks merits their consideration in pandemic strategy building.

The International Health Regulations (IHR) (4) constitute a global agreement among 196 signatory countries that represents the world’s coordinated effort to prepare for and limit the spread of epidemics. Established in 2005 under the auspices of the World Health Organization, IHR delineates a set of approaches that participating countries can use to strengthen key pandemic capacities, including capacities for surveillance, risk communications, human resources, laboratory testing, national legislation, coordination, response, and operational readiness (5). In 2014, multiple countries partnered to accelerate compliance with the 2005 IHR by developing the Global Health Security Agenda (GSHA) (6). However, despite the demonstrable relevance of NCDs to pandemic outcomes, neither IHR nor GHSA preparedness plans refer to NCD-related aspects of public health protection, potentially overlooking an important ingredient in global health security.

We posit that international pandemic prevention strategies, such as IHR and GHSA, can be improved by incorporating key NCD-related components. We identify 6 action areas in IHR and GHSA that are appropriate for integrating NCD-related objectives: surveillance, workforce development, laboratory systems, immunization, risk communication, and sustainable financing (3).

**Surveillance.** Surveillance, or the collection of population health data, is a core component of disease prevention and control. Including NCD-related data in GHSA surveillance initiatives provides several advantages to health security. First, it can support the development of population risk profiles, which can inform resource mobilization and health system planning (7–9). Second, it can improve the detection of the sequelae of infectious diseases, for example, HIV-associated NCD complications (10,11). Third, by signaling responsiveness and a long-term commitment to local health needs that is not limited to emergency events, it can broaden local support of GHSA/IHR priorities (12,13).

**Workforce development.** Workforce development refers to strengthening the skills of clinicians and other health professionals in supporting national public health priorities. Mechanisms for workforce development include initiatives such as the Field Epidemiology Training Program (14) and programs for training community health workers. The Field Epidemiology Training Program traditionally focuses on developing expertise in infectious disease epidemiology, but it can be adjusted to incorporate NCD-related curricula as well (15–17). Likewise, training programs for community health workers in countries whose populations have limited access to health care are well positioned to develop frontline workers who can bridge gaps in basic care between common NCD conditions and infectious disease prevention and control (18–20).

**Laboratory systems.** IHR and GHSA both emphasize the importance of establishing essential laboratory capacity in participating countries (21). Besides requiring assays for detection of pathogens commonly linked to epidemic diseases such as influenza, polio, HIV, tuberculosis, malaria, and salmonella, GHSA laboratory initiatives can allow for analysis of biomarkers of key NCD conditions. This capacity may facilitate whole-patient care while increasing economies of scale in laboratory processing.

**Immunization.** Vaccination against infectious pathogens that raise the risk of NCDs presents a unique opportunity for integrating global health security and NCD-related objectives. Vaccines for NCD risk reduction include, for example, those that protect against infection with human papillomavirus (a risk factor for cervical cancer) and hepatitis B (a risk factor for liver disease). Besides strengthening population health, vaccinations that reduce the risk of NCDs can support IHR and GHSA objectives by establishing vaccination channels among adults (22).

**Risk communication.** Risk communication is a valuable element of pandemic control (23). Including NCD-related information in emergency risk communication plans can improve response to infectious outbreaks in populations with comorbid conditions that make them vulnerable to infection.

**Sustainable financing.** Communicable and noncommunicable diseases increasingly depend on a shared health infrastructure (24,25). Shared infrastructure elements such as procurement channels can increase cost and operational efficiency. For example, the Pan American Health Organization Strategic Fund, a mechanism for procuring essential health supplies in the Pan American region, aggregates the supply of medications across the disease spectrum, increasing overall financial sustainability through economies of scale (26). Furthermore, NCD prevention approaches such as tobacco and alcohol taxation can be used to bolster the financial sustainability of health systems in low-income and middle-income countries (27–29).

The COVID-19 pandemic has amplified the importance of NCDs to outbreak preparedness and response. It has highlighted challenges experienced by health care systems worldwide in containing a severe infectious disease outbreak intensified by concurrent chronic health conditions. The aggravating effect of NCD comorbidities that disproportionately affect socioeconomically vulnerable groups has deepened equity concerns. Responding to the compounding burden of NCDs may improve readiness for public health emergencies.
